# The paradox of the life sciences: How to address climate change in the lab

**DOI:** 10.15252/embr.202256683

**Published:** 2023-02-02

**Authors:** Nikola Winter, Raphaël Marchand, Christian Lehmann, Lilian Nehlin, Riccardo Trapannone, Dunja Rokvić, Jeroen Dobbelaere

**Affiliations:** ^1^ Max Perutz Labs, Department of Biochemistry and Cell Biology University of Vienna Vienna Austria; ^2^ Faculty of Physics University of Vienna Vienna Austria; ^3^ Max Perutz Labs, Department of Structural and Computational Biology University of Vienna Vienna Austria; ^4^ Institute of Molecular Biotechnology of the Austrian Academy of Sciences Vienna Austria; ^5^ Vienna BioCenter PhD Program Doctoral School of the University of Vienna and Medical University of Vienna Vienna Austria; ^6^ Max Perutz Labs, Department of Microbiology, Immunobiology and Genetics University of Vienna Vienna Austria

**Keywords:** Economics, Law & Politics, History & Philosophy of Science, Methods & Resources

## Abstract

Addressing climate change and sustainability starts with individuals and moves up to institutional change. Here is what we as scientists in the life sciences can do to enact change.

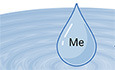

This commentary was inspired during a conference in a remote location over a discussion whether it was justified that one of the authors took a plane to get there. It quickly broadened into a general discussion of how to deal, as individuals and as scientists, with the dichotomy of studying the living world and the fact that the energy footprint of our work negatively impacts on the biosphere. Here, we discuss this issue from our experience as members of Climate@MaxPerutzLabs, an environmental grassroots group at the Vienna Biocenter.

Climate change is a systemic problem, and it requires a rapid and pervasive response across all sectors of society: government, business, non‐profit organizations, and education and research, down to each individual. Indeed, we cannot wait for others to solve this crisis; each of us has to take part in addressing climate change. We can reduce our personal impact on greenhouse gas (GHG) emissions and environmental degradation, and we can advocate and take leadership to promote systemic change at whatever position we are within our institutions and professional networks. As academic researchers, we can work with the management to set standards for more sustainable research and show by example that these are practicable. As research institutes and universities are interlinked with biomedical and pharmaceutical companies, we can also hope for a multiplier effect via collaborative and supply chain networks and via the students we train to spread an attitude of environmental awareness into companies and other institutions.

Biomedical and molecular biological research has led to great achievements and benefits, but it comes at a cost.

## A critical time

In 2019, the average GHG emissions per capita in Europe were 8.1 t of carbon dioxide equivalents (CO_2_eq); this dropped to 7.4 t CO_2_eq in 2020 (OECD.Stat, Data extracted on 26 Jan 2023). However, in order to limit global warming to 1.5°C, carbon emissions must further drop to net zero by 2050. This would require a yearly decrease comparable to the drop observed from 2019 to 2020 during the COVID‐19 pandemic (Friedlingstein *et al*, 2022).

Estimates for the yearly, work‐related carbon footprint of a life scientist range from ca. 4 to 15 t CO_2_eq.

Nonetheless, in 2021, carbon emissions went back to pre‐pandemic levels (Tollefson, 2021). At the current pace, the remaining ‘carbon budget’ buffer to restrict global warming to 1.5°C will be gone within 9 years (Friedlingstein *et al*, 2022). The global mean temperature in 2022 was 1.15°C above the pre‐industrial average and is expected to further increase to an average global temperature increase of 2.8°C by the end of this century (UNEP Gap Emissions Report, 2022) if we continue with business as usual. Substantial reductions of GHG emissions during this decade are therefore essential to meet the targets ratified by 193 states and the EU in the Paris Agreement.

## The paradox

As life scientists, we do not want to harm life, but, by our very actions, we inevitably do. Biomedical and molecular biological research has led to great achievements and benefits, but it comes at a cost. Air travel, on‐demand delivery of consumables and reagents, energy and resource‐intensive infrastructure, single‐use consumables and plastics: all of this entails GHG emissions, freshwater consumption, soil depletion, and accumulation of waste and hence impacts on biodiversity (Pörtner *et al*, 2021; Bull *et al*, 2022). We are part of the problem, but we can also be part of the solution. There are ways to substantially decrease the consumption of resources and energy without impacting on the quality of research. Slow science and a focus on quality instead of quantity could help improve sustainability.

Surprisingly, carbon accounting is still not widely established in academic institutions. EU regulations require larger companies to publish regular reports on the social and environmental impacts of their activities (https://finance.ec.europa.eu/capital‐markets‐union‐and‐financial‐markets/company‐reporting‐and‐auditing/company‐reporting/corporate‐sustainability‐reporting_en#standards), but there are no similar requirements or standards for universities or publicly funded research institutions. Reports from 25 European universities reveal a huge variability in regard to the estimated carbon footprints, from less than 1 to 32 t CO_2_eq per employee (ALLEA, 2022), as these reports vary greatly in scope and are therefore difficult to compare (Borgermann *et al*, 2022). In particular, scope 3 or indirect emissions not related to purchased energy are not easy to quantify and therefore only partially included in many GHG balances. Notably, they can account for up to 90% of total GHG emissions of universities (https://pdf.ethz.ch/eth‐whitepaper‐net‐zero‐by‐2030/67177854).

Estimates for the yearly, work‐related carbon footprint of a life scientist range from ca. 4 to 15 t CO_2_eq (ALLEA, 2022). The upper estimate is consistent with our own calculations for 2019 using the ClimCalc tool (https://nachhaltigeuniversitaeten.at/arbeitsgruppen/co2‐neutrale‐universitaeten/), which does not include the footprint of consumables, equipment, and construction and building maintenance. Thus, even the highest number is probably still an underestimation. The field is still learning about the extent of contributing factors, but as of now, the GHG emissions of molecular biological research mainly fall into four categories: real estate and infrastructure; travel and commuting; the production, transport, and disposal of equipment, consumables, chemicals, and reagents; and the energy usage by heating, ventilation, and air conditioning (HVAC) of laboratory buildings (Kitzberger *et al*, 2022) and electrical devices. Box 1 compiles some best practice examples of what can be done on the individual or lab level to reduce GHG emissions across these categories. Many of these measures also result in financial savings, which should make them economically attractive for management.

Box 1 **What can be done on the lab level.**

- Make sure your research is of the highest quality: experiments that cannot be reproduced or interpreted because they were not planned carefully enough are a waste of resources and time. Determine in advance the required sample size to obtain statistically meaningful data. Make sure your results are reproducible by documenting your experiments diligently; this starts, as trivial as it might seem, with labeling your samples properly. Diligent planning of experiments also helps prevent unnecessary waste.
- Teach and train your students and new lab members in sustainable research practices.
- Optimize your travel. Avoid flights if possible. Ask yourself whether it is really necessary to go to a specific conference in person. You may also refrain from using flights when traveling a distance less than 1,000 km or when the destination can be reached by train or bus within 12 h (https://unter1000.scientists4future.org/best-practice-administration/). If flying is necessary, avoid stopovers. When commuting to work, use public transport or a bike or walk instead of using a car. Consider moving closer to your institute. To instigate systemic change, ask your institution to enact a flight policy, to support cycling – for instance, by providing enough safe bike parking options, showers, and changing rooms for cyclists – and to support the use of public transport by subsidizing tickets for employees.
- Join a lab certification program such as the Laboratory Efficiency Assessment Framework (LEAF: https://www.environment.admin.cam.ac.uk/green-labs/leaf). Developed by members of the University College London, this tool can help reduce the carbon footprint of your work. It offers a carbon and a cost calculator and comes in the form of a competition. You can achieve bronze, silver, and gold in different categories: ventilation, equipment, procurement, waste, samples, chemicals, and research quality. Institutes can subscribe annually, and labs can participate individually. Alternative programs are the My Green Labs Certification (https://www.mygreenlab.org/green-lab-certification.html) and GES 1point5 (https://apps.labos1point5.org/ges-1point5).
- Products and consumables make up a large part of a lab's environmental footprint. For single‐use consumables, we consider the 5‐R‐rule: refuse, reduce, reuse, repurpose, and recycle. Reduce the use of reagents too. Reactions can often be done in smaller volumes than suggested by the manufacturer. Share chemicals and equipment with other labs. Coordinate your orders to reduce transport. You can agree with companies on weekly instead of daily shipments. Some suppliers offer setting up vending machines on campus with the most commonly ordered reagents, which helps reduce transport. Talk to company representatives about the climate strategy of their enterprise and ask for more sustainable alternatives to standard products. Buy products from companies that have a clear and detailed sustainability plan and offer certified products.
- Close the sash of laboratory fume hoods and turn them off when not in use. Fume hoods can consume 3 to 3.5 times more than ULT freezers when the sash is not closed because they suck in conditioned air from the surrounding; a laboratory's HVAC system has air flow rates two to four times higher than in office buildings and is the single most energy‐intensive service in laboratory buildings (Kitzberger *et al*, 2022).
- A conventional ultra‐low temperature (ULT) freezer uses approximately 20 kWh of energy per day, which is equivalent to the energy use of the average US household. You can reduce its energy usage in many ways: regularly sort out unneeded samples and share freezer space with other labs to reduce the overall number of freezers needed. Keep an updated inventory list ‐ quick access to your samples greatly reduces the time when the freezer door is open. Open doors and high ambient humidity lead to frost buildup, which may even stop the door from closing and cause stress on the gaskets. It is therefore recommended to remove ice and frost twice a year. Dust accumulation on the filter increases energy consumption by approximately 14% and even 25% if coupled with dust accumulation on the condenser fins (Gumapas & Simons, 2013). Filters should be cleaned every 3 months. Ambient temperature, spacing, and age of ULT freezers additionally affect their energy consumption. Finally, ULT freezers can safely be operated at −70°C, instead of −80°C, which can reduce energy consumption by 10–30% (Faugeroux, 2016). Implement a “lab exit protocol” for members that leave the lab to free up freezer space and avoid old samples no one knows what they contain. You may also join the Freezer Challenge, an international competition organized by two nonprofits to make cold storage more energy‐efficient, save costs, and at the same time make samples more accessible (https://www.freezerchallenge.org/).
- Run autoclaves, sterilizing ovens, and dishwashers only when full.
- Switch off laboratory equipment such as water baths, cooled centrifuges, and light when not in use. Do not set thermocyclers to hold samples at 4°C, especially when running overnight. A hold at 12°C is sufficient, unless your reaction is contaminated with nuclease. Establish and communicate a “last to leave” protocol in your lab so that the last person switches off equipment and puts samples into fridges and freezers (https://greenlabsaustria.at/how-to-get-started/).
- Stickers can help inform and remind colleagues of sustainable practices.
- Avoid sending large e‐mail attachments – better share files via download link.
- Replace toxic chemicals with less toxic ones if possible.
- Be creative. In our institute, some groups still prefer to use ethidium bromide agarose gels, which end up as toxic waste incinerated at high temperatures. We found that air drying of ethidium bromide gels before disposal reduces their weight by >95%, which decreased our amount of toxic waste by up to 80%.
- Reduce plastic waste by planning your experiments well, replacing single‐use plastics with glassware, and reusing plastic where possible (Bowler, 2022; preprint: Farley & Nicolet, 2022). Some producers also offer to take back their used consumables (e.g., tissue culture media bottles, pipette tip boxes, and gloves). Depending on your local infrastructure and regulations, your lab plastic may be eligible for recycling if contamination can be excluded.

## Individual initiative needed

**Figure 1:**
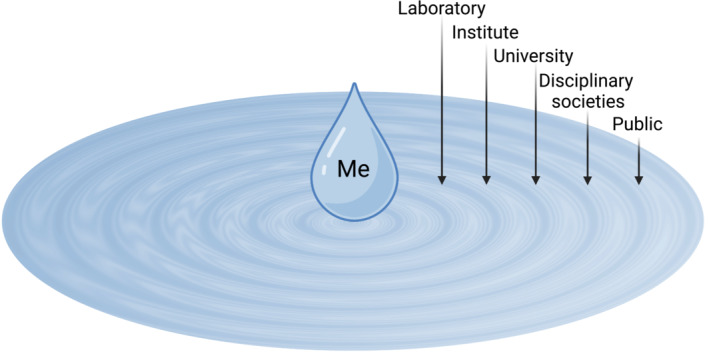
Spheres of influence on environmental sustainability for academics. Created with BioRender.com and Adobe Photoshop.

The 2015 Paris Agreement, IPCC's Special Report on Global Warming in 2018 (https://www.ipcc.ch/sr15/) and national declarations of carbon neutrality targets have set the stage for addressing climate change globally. Research institutes and universities in Europe including BOKU Vienna, ETH Zürich, the École Polytechnique Fédérale de Lausanne, University of Copenhagen, and universities in the UK such as Oxford, Cambridge, and University College London, have put a high priority on sustainability. They established sustainability offices and started assessing their carbon footprint and drafted roadmaps to achieve carbon neutrality. From these examples, we can learn that individuals in administration, management and the governing body can significantly accelerate institutional change. But, individual researchers within their institutions and professional networks have also spheres of influence. They can change their personal practices, make environmental sustainability an integral part of their teaching, encourage their colleagues within and beyond their own groups and institutions to adopt more sustainable practices, and engage in public discourse (Fig 1).

On the individual level, we can only do so much, but together, we can achieve a change in institutional culture, such as research groups, institutes, universities, or learned societies. A lesson we learned is that stimulating or facilitating institutional change is at least as much a matter of diplomacy as of technical solutions. We will briefly highlight two critical characteristics of the academic system that affect the success of stepping up from individual initiatives to collective action and then institutionalization: high staff turnover and hierarchy.

On the individual level, we can only do so much, but together, we can achieve a change in institutional culture, such as research groups, institutes, universities, or learned societies.

As academics, we enjoy considerable autonomy in our work, which allows us to a certain degree to pioneer more sustainable practices, conduct pilot studies, and collect data. However, the continuation of these practices is not guaranteed when engaged persons leave the institution. Therefore, ways of doing research more environmentally sustainable need to be established institutionally. This means more than decreeing do's and don'ts: it implies a cultural change that involves raising awareness, negotiation, education, and training to arrive at and align with a common vision and mission. Once this institutional culture is established, the spirit, practices, and regulations are passed on to newcomers, ensuring that sustainable practices continue even despite high staff turnover. The latter has the positive effect that it helps spread experience and awareness for environmental sustainability between labs around the globe.

**Figure 2:**
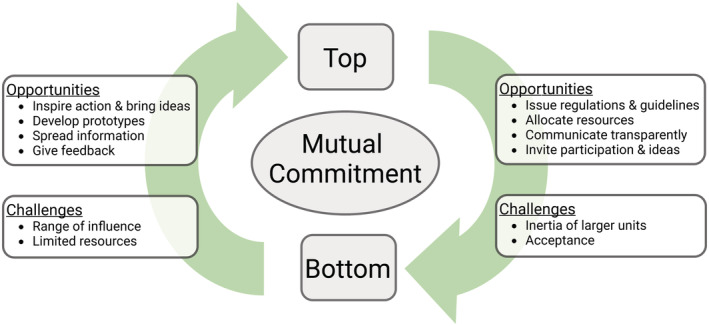
Our vision for synergistic collaboration across hierarchies at various institutional scales. Created with BioRender.com.

Academia is largely hierarchically organized, which implies an asymmetric distribution of power. Change of practices can be decreed (top–down) or demanded (bottom–up). Either alone might create tensions or even trigger rejection. But, an interdigitation of top–down and bottom–up approaches based on mutual commitment to a common goal of environmentally sustainable practices has proven successful for institutional change (see Box 2 for best practice examples). Each side has unique opportunities and challenges which, when matched, can be a recipe for success (Dobbelaere *et al*, 2022; Fig 2).

Box 2 **Seven examples for fruitful collaboration between the top and bottom.**

- **Centre for Global Change and Sustainability** at the University of Natural Resources and Life Sciences (BOKU) Vienna https://boku.ac.at/en/nachhaltigkeit. In 2010, the rectorate established the Centre for Global Change and Sustainability to promote sustainability and facilitate networking to create a point of contact and to raise awareness. In 2012, together with the University of Graz, BOKU initiated the Alliance of Sustainable Universities in Austria and was one of the first universities in Austria to develop a sustainability strategy in a broad, participatory process with 140 participants and support from the rectorate. Since 2019, BOKU has published an annual, externally certified Sustainability Report in accordance with internationally applicable Global Reporting Initiative (GRI) standards.
- **The Max Planck Sustainability Network** (MPSN) www.nachhaltigkeitsnetzwerk.mpg.de. Founded in 2019, the MPSN is a grassroots initiative within the Max Planck Society (MPG). Several hundred members from 65 different institutes are organized in individual sustainability groups focusing on topics such as energy, mobility, supplies & waste, and biodiversity & food (Fardet *et al*, 2020), with the common goal to make the MPG and science in general more ecologically sustainable. The network is coordinated and represented by a steering committee, which is elected by representatives of the sustainability groups on an annual basis. In 2021, MPSN published a Catalog of Recommendations for Sustainability in the Max Planck Society (https://www.nachhaltigkeitsnetzwerk.mpg.de/doi-2021-care).
- **Dunn School Green Group** (Environmental committee at the Sir William Dunn School of Pathology at University Oxford) https://mobile.twitter.com/dunn_gogreen. Established in March 2020, this is an active grassroots group including PIs that is well‐connected with the Oxford University sustainability team in the central administration. In 2022, the group received two awards for their efforts to reduce laboratory carbon emissions: the High Sheriff's Climate Action Heroes Award and the Vice Chancellor's Environmental Sustainability Award.
- **ETH Zürich's Air Travel Project** https://ethz.ch/en/the-eth-zurich/sustainability/eth-sustainability/air-travel.html. Launched in 2017 by the executive board, the Air Travel Project is a best practice example for a comprehensive approach to reduce air travel without a negative effect on scientific excellence. It is based on participative decision‐making and transparent communication. This model is the result of an institutional learning process that started in 2006 after a less successful top–down‐only approach (Kreil, 2021).
- **EPFL Sustainability** (at the École Polytechnique Fédérale de Lausanne) www.epfl.ch/about/sustainability. In 2019, upon the initiative of employees, the EPFL directorate set up a task force to establish the Campus Climate Plan, a roadmap for the years 2021–2030. In order to define targets and measures to reach the defined goals, a participatory approach with workshops and working groups was held, and a vice presidency for responsible transformation at EPFL was appointed to accompany this process. The EPFL sustainability unit has an office and currently consists of 13 members, as well as five further members embedded in the Schools of Life Sciences, Basic Sciences, and Engineering.
- **Laboratory Efficiency Assessment Framework** (LEAF) at the University College London (UCL) www.ucl.ac.uk/sustainable/make-your-lab-sustainable-leaf. UCL established a position to explore, quantify, and communicate various aspects of environmental sustainability, with a focus on efficiency and reproducibility. The result was LEAF, a tool that is now shared with more than 80 institutions in the UK, Europe, and abroad. UCL is also a part of **LEAN**, the Laboratory Efficiency Action Network (www.lean-science.org), with the goal to share best practice examples from daily actions to institutional strategy or policy change.
- **Green EMBL** https://www.embl.org/about/sustainability/. At the European Molecular Biology Laboratory, an active grassroots group run by the EMBL Staff Association and supported by the direction had long been established. In 2019, management appointed a Sustainability Officer and commissioned a Materiality Assessment that was taken as the basis for a comprehensive Sustainability Strategy. EMBL's Sustainability Office publishes an annual Sustainability Report.

… if employees are invited to contribute to the decision‐making process and informed about the motivation underlying new regulations, they feel heard and respected and can even give valuable feedback.

As they are directly immersed in the day‐to‐day workings of an institution, employees can identify potential for improvement that might not be obvious to management. Management may tap this knowledge by inviting suggestions for optimization. In addition, grassroots groups are more agile than big institutions. This makes it easier for them to explore and test new procedures and to develop prototypes. Successful pilot studies may then be taken to scale and institutionalized by management.

A common problem is that directives may be perceived as imposing on individual freedom, an unnecessary increase of work or even endangering freedom of research. Yet, if employees are invited to contribute to the decision‐making process and informed about the motivation underlying new regulations, they feel heard and respected and can even give valuable feedback. They can then spread their insight in their informal networks with colleagues, which increases the understanding and acceptance for new directives. Vice versa, if employees collectively demand change, management will feel more confident to take action. These synergies apply across scales: from the individual research group to the institute and to the university. Box 3 gives a starter kit for people who want to get active but are new to the field of sustainability in research institutes.

Box 3 **How to get started.**

Explore, get informed, raise your own awareness. Here are some hubs for gathering information and starting to network.

- The ALLEA Report “Climate Sustainability in the Academic System in Europe and Beyond” (ALLEA, 2022) published in 2022 by the German Young Academy.
- The Sustainable European Laboratories network (https://sels-network.org/) consists of individuals and research groups in Europe. It offers information and best practice examples, helps connect people, assists individuals who want to start their own initiative, and aims to influence policymakers and key stakeholders.
- There are various sustainable life science laboratory initiatives at individual universities: University of Boulder Colorado (https://www.colorado.edu/ecenter/greenlabs), Oxford University (https://sustainability.admin.ox.ac.uk/labs), Harvard University (https://green.harvard.edu/programs/green-labs), University of Exeter (https://www.exeter.ac.uk/about/sustainability/sustainablelabs), University of Pennsylvania (https://sustainability.upenn.edu/green-labs-guide), University of Queensland (https://sustainability.uq.edu.au/get-involved/green-labs-program),
- and a collection of links to Green Labs Programs of Universities in the USA (https://www.colorado.edu/ecenter/greenlabs/campus-resources/links-univ-green-labs-programs).
- A trustworthy report clearly states its scope and limitations (state of the art are the “GRI standards”; https://www.globalreporting.org/standards/). Examples are the University of Cambridge Annual Report 2020–21 (www.environment.admin.cam.ac.uk/Annual-Report), the Max Delbrück Center for Molecular Medicine Berlin Greenhouse Gas Balance 2019/2020 (https://www.mdc-berlin.de/media/41956), the ETH Zürich 2019/2020 Report (https://ethz.ch/content/dam/ethz/main/eth-zurich/nachhaltigkeit/Berichte/Nachhaltigkeitsbericht/ETHzurich_Sustainability_Report_2019_2020_web.pdf), and the ETH's Whitepaper that addresses the impact of Scope 3 emissions (https://ethz.ch/en/the-eth-zurich/sustainability/net-zero.html).
- I2SL (The International Institute for Sustainable Laboratories; www.i2sl.org) is a non‐profit organization that provides education and information to the sustainable laboratory community. I2SL organizes an annual conference and workshops and hosts working groups and local chapters. The website offers diverse resources for free. Individuals and institutions can apply for membership for additional benefits. I2SL is sponsored by companies.
- My Green Lab® (www.mygreenlab.org), is a US non‐profit organization that provides information, hosts a podcast and a blog, offers programs like the ambassador program, the freezer challenge, a certification for labs and a certification for products (ACT® label), organizes summits, and offers courses. It is sponsored by companies.
- Labconscious® (www.labconscious.com) is a blog operated by a company that produces reagents. It offers tips on how to reduce laboratory waste, use green chemistry, conserve water and save energy, and advertise green lab supplies and laboratory equipment. It also provides a list of green lab groups to facilitate networking.
- The Podcast “The Caring Scientist: Mission Sustainable” was launched in April 2021 and systematically covers various aspects of making labs more sustainable.

**Connect with others**

- Talk to colleagues, students, and superiors.
- Join a local, regional, or international group.
- Start a group in your institute.

**Take measures**

- In your lab (see Box 2).
- Communicate the topic of environmental sustainability in the lab (teach students, inform your colleagues, and talk about it on conferences).
- If your institute has no sustainability strategy, ask for one.
- Make a survey to collect more information about your institute or university. This is a tool to assess the willingness to support measures, it can help evaluate past measures, and it may be the first step to recruit members for a grassroots group.

**Keep on going**

Be patient but persistent – even small steps are a contribution, substantial change takes its time. If you work on establishing operational changes, aim for institutional implementation of these measures so that their continuation is guaranteed even if you leave the institute at some point.

## Examples

Here are three examples of such interactions at our University of Vienna. In 2021, the rectorate for infrastructure appointed an advisory board consisting of members from the senate, the faculty, and the management of postdocs, admins, technicians, and students. The board accompanied the appointment of a sustainability coordinator, the proclamation of a sustainability strategy, a roadmap to reach carbon neutrality in 2030, and carbon balance in 2022. Now subgroups, including members from grassroots initiatives, work on the details to apply the roadmap to their area of responsibility, for example, in research labs.

The Switch It Off Ticket: The University of Vienna's infrastructure department invites employees to submit ideas for energy‐saving. The suggestions are treated, like other technical orders, via a ticket system by the respective local person in charge and in parallel communicated to the sustainability office.

Spearheaded by Green Labs Austria, another active grassroots group, we quantified how much of the laboratory plastic waste – that cannot be reduced or replaced by glassware – could be recycled (https://greenlabsaustria.at/recycling‐lab‐plastic/). We designed and tested a workflow to separately collect different kinds of plastics while avoiding contamination, as this would prevent plastic recycling. Currently, management assesses the feasibility for university‐wide scaleup of plastic recycling in labs.

## Conclusion

Our current research practices in the life sciences are not aligned with environmental sustainability. As individuals embedded in our institutions, we can however contribute to the needed systemic shift by changing our practices and by acting as role models for others, including students, who will in turn disseminate this attitude and thereby accelerate social change. With a general rise in awareness of the climate crisis, the past years have seen a steep increase in initiatives toward more sustainable research both from institutions and grassroots initiatives, and this trend will hopefully persist until environmental sustainability has become an integral feature of research.

Adopting sustainable research practices […] should naturally be an integral feature of research such as following health and safety regulations and ethical standards.

Adopting sustainable research practices must not be perceived as an additional burden or even a hindrance for scientific excellence and success but, on the contrary, it should naturally be an integral feature of research such as following health and safety regulations and ethical standards. As we were inspired by others, we now encourage our colleagues to take initiative in their areas of influence and to team up. Let us act together to solve the paradox of the life sciences. It is part of our mission, as life scientists, to protect life and deliver a better planet to future generations.

## Disclosure and competing interests statement

The authors declare that they have no conflict of interest.

## Supporting information


