# Supplementary material for: The paradox of the life sciences: How to address climate change in the lab
Source: EMBO Rep. 2023 Feb 2;24(3):e56683. doi: 10.15252/embr.202256683 (PMC9986813; doi:10.15252/embr.202256683)
Supplement: Supplementary file 1 [file EMBR-24-e56683-s001.pdf]

The paradox of the Life Sciences

Nikola Winter, Raphaël Marchand, Christian Lehmann, Lilian Nehlin, Riccardo Trapannone, Dunja Rokvić, and Jeroen Dobbelaere
DOI: 10.15252/embr.202256683

Corresponding author(s): Nikola Winter (nikola.winter@univie.ac.at)

Review Timeline:

Submission Date:	16th Dec 22
Accepted:	17th Jan 23

Editor: Holger Breithaupt

Transaction Report: This article was editorially reviewed.
